# Negative lymph node count is a significant prognostic factor in patient with stage IV gastric cancer after palliative gastrectomy

**DOI:** 10.18632/oncotarget.17430

**Published:** 2017-04-26

**Authors:** Changhua Zhuo, Mingang Ying, Ruirong Lin, Xianyi Wu, Shen Guan, Chunkang Yang

**Affiliations:** ^1^ Department of Gastrointestinal Surgical Oncology, Fujian Provincial Key Laboratory of Tumor Biotherapy, Fujian Cancer Hospital & Fujian Medical University Cancer Hospital, Fuzhou 350014, China

**Keywords:** gastric cancer, palliative resection, negative lymph node, survival analysis, prognostic factor

## Abstract

Negative lymph node (NLN) count has been validated as a protective predictor in various cancers after radical resection. However, the prognostic value of NLN count in the setting of stage IV gastric cancer patients who have received palliative resection has not been investigated. Surveillance, Epidemiology, and End Results Program (SEER)-registered gastric cancer patients were used for analysis in this study. Kaplan-Meier survival curves and multivariate Cox proportional hazards model were used to assess the risk factors for patients’ survivals. The results showed that NLN count and N stage were independently prognostic factors in patients with stage IV gastric cancer after palliative surgery (P< 0.001). X-tile plots identified 2 and 11 as the optimal cutoff values to divide the patients into high, middle and low risk subsets in term of cause-specific survival (CSS). And NLN count was proved to be an independently prognostic factor in multivariate Cox analysis (P< 0.001). The risk score of NLN counts demonstrated that the plot of hazard ratios (HRs) for NLN counts sharply increased when the number of NLN counts decreased. Collectively, our present study revealed that NLN count was an independent prognostic predictor in stage IV gastric cancer after palliative resection. Standard lymph node dissection, such as D2 lymphadectomy maybe still necessary during palliative resection for patients with metastatic gastric cancer.

## INTRODUCTION

Gastric cancer is one of the most common and deadliest malignancies globally, especially in eastern Asian [1, 2]. For patients with resectable gastric cancer, surgical resection is the only potentially curative therapeutic modality, and the lymph node (LN) status turns to be the strongest prognostic indicator for survival after gastrectomy [3, 4]. According to the current tumor-node-metastasis (TNM) staging system for gastric cancer of the Union for International Cancer Control/American Joint Committee on Cancer (UICC/AJCC) and Japanese Gastric Cancer Association (JGCA) guideline, a minimum of 15 dissected LNs should be collected from gastric cancer samples for accurate histological examination and proper N stage assessment [5-7]. The number of retrieved LN counts was also validated as predictor after radical resection [6, 8, 9].

The proportion of stage IV cancer increased significantly during the last two decades and comprised more than 40% of total cases [10, 11]. The prognosis of gastric cancer patients with distant metastases is poor, and the 5-year overall survival (OS) rate rarely exceeds 5% if received palliative chemotherapy only [12]. Half of the patients with advanced gastric carcinoma will develop severe tumor-related complications, such as bleeding, obstruction and perforation, for the rest of their lives. Palliative surgery was recommended in this situation [11-13].

With advances in medical technologies, surgical interventions for patients with distant metastases are gaining more attention, and it has scientifically proved to have survival benefit [14, 15]. Meta-analysis showed that gastric resections were associated with a 2.5-fold higher OS rate than conservative treatment [11]. However, the clinical importance of regional LN in stage IV gastric is not fully understood.

The concept of negative lymph node (NLN) has recently attracted attention as a prognostic factor in colon [16, 17], gastric [9, 18], esophageal [19], and cervical [20] cancer. However, the correlation between NLN count and prognosis in patients with stage IV gastric cancer is not clear. Thus, the aim of this retrospective study was to explore the effect of NLN counts on the survival outcomes in patients with stage IV gastric cancer after palliative resection.

## RESULTS

### Patient characteristics

**Figure 1:**
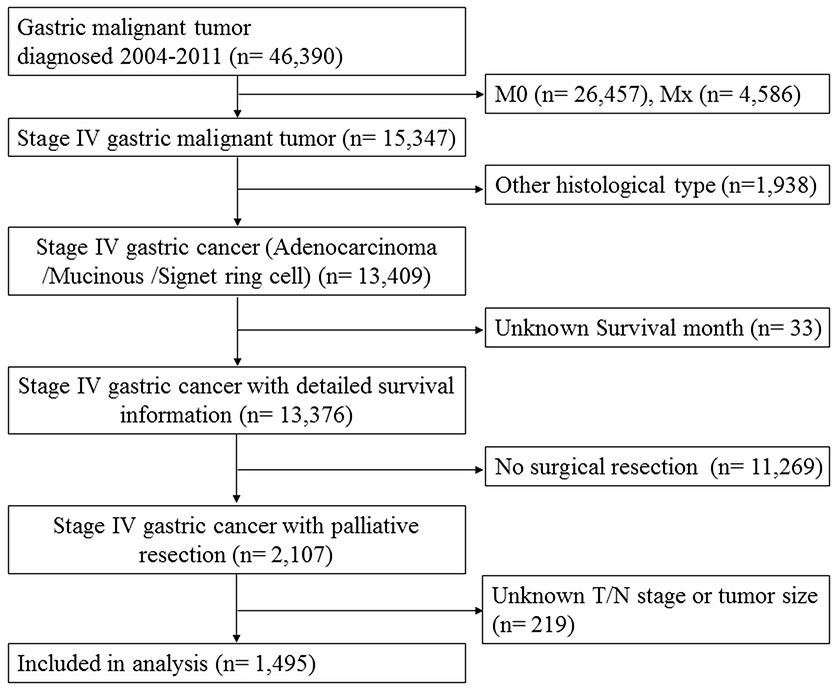
The flow chart of eligible patient selection in the present study Surveillance, Epidemiology, and End Results (SEER)database collects incidence and survival data of gastric cancer from population-based cancer registries covering 28 percent of US population. Refer to the section of Patients’ Characteristics for study inclusion criteria.

A total of 1,495 patients with stage IV gastric cancer underwent palliative resection were identified from Surveillance, Epidemiology, and End Results (SEER) database between 2004 and 2011. The flow chart of the study was depicted in Figure 1. Of all patients, 144 (9.63%) were confirmed with no primary lymph node metastases (pN0), while 1,351 (90.37%) with primary lymph node involved (pN+). A detailed description of the associations between N stage and clinicopathological characteristics were presented in Table 1. As anticipated, patients of advanced T stage, poor differentiation and mucinous /signet ring cell had higher percentage of lymph node involved (*P*< 0.05).

**Table 1:** Demographic and associations between lymph node status and clinicopathological characteristics of patients with stage IV gastric cancer

Characteristic	pN0 (n= 144) No. (%)	pN+ (n = 1351) No. (%)	*χ*^2^	*P*
Sex			0.027	0.871
Male	90 (62.5)	835 (61.8)		
Female	54 (37.5)	516 (38.2)		
Age			1.022	0.312
≤60	43 (29.9)	460 (34.0)		
>60	101 (70.1)	891 (66.0)		
Race			6.022	0.049
Caucasian	108 (75.0)	885 (65.5)		
Black	12 (8.3)	195 (14.4)		
Other*	24 (16.7)	271 (20.1)		
Pathological grading			14.580	0.001
High/ Moderate	48 (33.3)	267 (19.8)		
Poor/ Anaplastic	91 (63.2)	1038 (76.8)		
Unknown^*^	5 (3.5)	46 (3.4)		
Histological type			6.792	0.034
Adenocarcinoma	111 (77.1)	897 (66.4)		
Mucinous /Signet ring cell	33 (22.9)	354 (33.6)		
T stage			29.940	< 0.001
T1-2	74 (51.4)	409 (30.3)		
T3	46 (31.9)	494 (36.6)		
T4	24 (16.7)	448 (33.2)		
No. of LNs retrieved, mean (range)	10.40 (1- 46)	16.49 (1- 88)		< 0.001
NLN counts, mean (range)	10.40 (1- 46)	6.28 (0- 81)		< 0.001

^*^ Other includes American Indian/Alaska native, Asian/Pacific Islander, and unknown. Abbreviation: pN0: no primary lymph node metastases; pN+: primary lymph node involved; LNs: lymph nodes. One-way ANOVA analysis was used. *P*-value <0.05 was considered to be statistically significant.

### Identification of optimal cutoffs of NLN in term of patients’ survival

**Figure 2:**
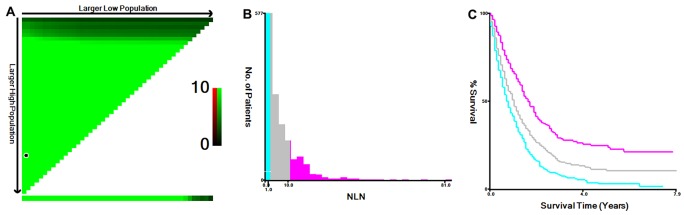
Cutoff value for NLN counts calculated using the X-tile program The X-tile program was utilized to calculate the optimal cutoff value for the negative lymph node (NLN) count. The entire population was divided into the training and validation sets based on the patient survival data. X-tile plots of training sets are shown in the upper-left quartile, with plots of the matched validation set in the small long strip (on the bottom X-axis). The black dot in the validation set represents the exact cutoff value for the NLN count **(A)** The entire cohort was divided into low (blue), middle (grey), and high (pink) NLN count groups based on the cutoff value (2 and 11), as shown in the histogram **(B)** Kaplan-Meier plots were generated based on the cutoff values (χ^2^= 77.318, *P< 0.001*) **(C)**.

The 5-year cause-specific survival (CSS) was 11.0% in this study cohort. NLN count was first treated as continuous variable, and it was validated as prognostic factor by univariate Cox regression analysis (hazard ratio (HR) = 0.961, 95% confidence interval (CI): 0.952-0.970, *P*< 0.001). Then, the X-tile program was utilized to divide the patients into high, middle and low risk subgroups with cutoff value of 2 and 11 in terms of 5-year CSS (Figure 2). The 5-year CSS rate for patients with 0-1, 2-10 and ≥11 were 3.2%, 10.8% and 24%, respectively (*χ*^2^= 77.318, *P*< 0.001). Specifically, there was an absolute 20.8% increase in 5-year CSS rate if ≥ 11 NLNs were retrieved compared with those who had 0-1 NLN.

### Prognostic impact of NLN counts

The numbers of NLN counts and other clinicopathological parameters, including sex (*P*= 0.005), grade (*P*= 0.013), histological type (*P*= 0.017), T stage (*P*< 0.001), and N stage (*P*< 0.001), were statistically significant variables in univariate analysis. They were further investigated by multivariate Cox regression analysis. The results demonstrated that only T stage (T3, HR= 1.098, 95% CI: 0.941-1.281, T4, HR= 1.343, 95% CI: 1.143- 1.577, *P*= 0.001, T1-2 as reference), N stage (N1, HR= 1.285, 95% CI: 0.965-1.713, N2, HR= 1.558, 95% CI: 1.187-2.045, N3, HR= 1.775, 95% CI: 1.373-2.296, *P*< 0.001, N0 as reference), and NLN count (2-10, HR= 0.762, 95% CI: 0.660-0.880, ≥11, HR= 0.525, 95% CI: 0.437- 0.632, *P*<0.001, 0-1 as reference) were significantly correlated with 5-year CSS (Table 2).

**Table 2:** Univariate and multivariate survival analyses for evaluating the influence of the number of NLNs retrieved on CSS in stage IV gastric cancer

Variables	5-year CSS rate	Univariate analysis		Multivariate analysis	
		χ^2^	*P*	HR (95% CI)	*P*
Sex		7.816	0.005		0.157
Male	15.9%			Reference	
Female	5.1%			1.098 (0.965- 1.249)	
Age		2.732	0.098		
≤ 60	13.7%				
> 60	10.4%				
Race		0.998	0.607		
Caucasian	12.1%				
Black	14.6%				
Others	9.1%				
Grade		8.734	0.013		0.344
High/ Moderate	12.2%			Reference	
Poor/ Anaplastic	11.2%			1.086 (0.924- 1.276)	0.319
Unknown	18.3%			0.888 (0.615- 1.283)	0.528
Histological type		8.155	0.017		0.582
Adenocarcinoma	13.5%			Reference	
Mucinous/ signet ring cell	7.4%			1.040 (0.905- 1.194)	
Tumor size (cm)		2.290	0.130		
< 5	12%				
≥ 5	11.7%				
T Stage		29.484	< 0.001		0.001
T1-2	18.3%			Reference	
T3	7.9%			1.098 (0.941- 1.281)	0.235
T4	6.0%			1.343 (1.143- 1.577)	< 0.001
N stage		57.607	< 0.001		< 0.001
N0	32.7%			Reference	
N1	18.6%			1.285 (0.965- 1.713)	0.087
N2	13.3%			1.558 (1.187- 2.045)	0.001
N3	4.6%			1.775 (1.373- 2.296)	< 0.001
LNs retrieved		0.542	0.461		
< 15	12.3%				
≥ 15	11.4%				
NLN counts		77.318	< 0.001		< 0.001
0-1	3.2%			Reference	
2-10	10.8%			0.762 (0.660- 0.880)	< 0.001
≥ 11	24.0%			0.525 (0.437- 0.632)	< 0.001

Abbreviation: CSS: cause-specific survival; HR: hazard ratio; 95% CI: 95% confidence interval; T stage: tumor infiltration depth; N stage: lymph node status; LNs: lymph nodes; NLN: negative lymph node. Kaplan-Meier survival curves and Log-rank tests were used to identify the univariate risk factors. Cox regression model was used to perform multivariate analysis. *P*-value <0.05 was considered to be statistically significant.

### The risk score of NLN counts

**Figure 3:**
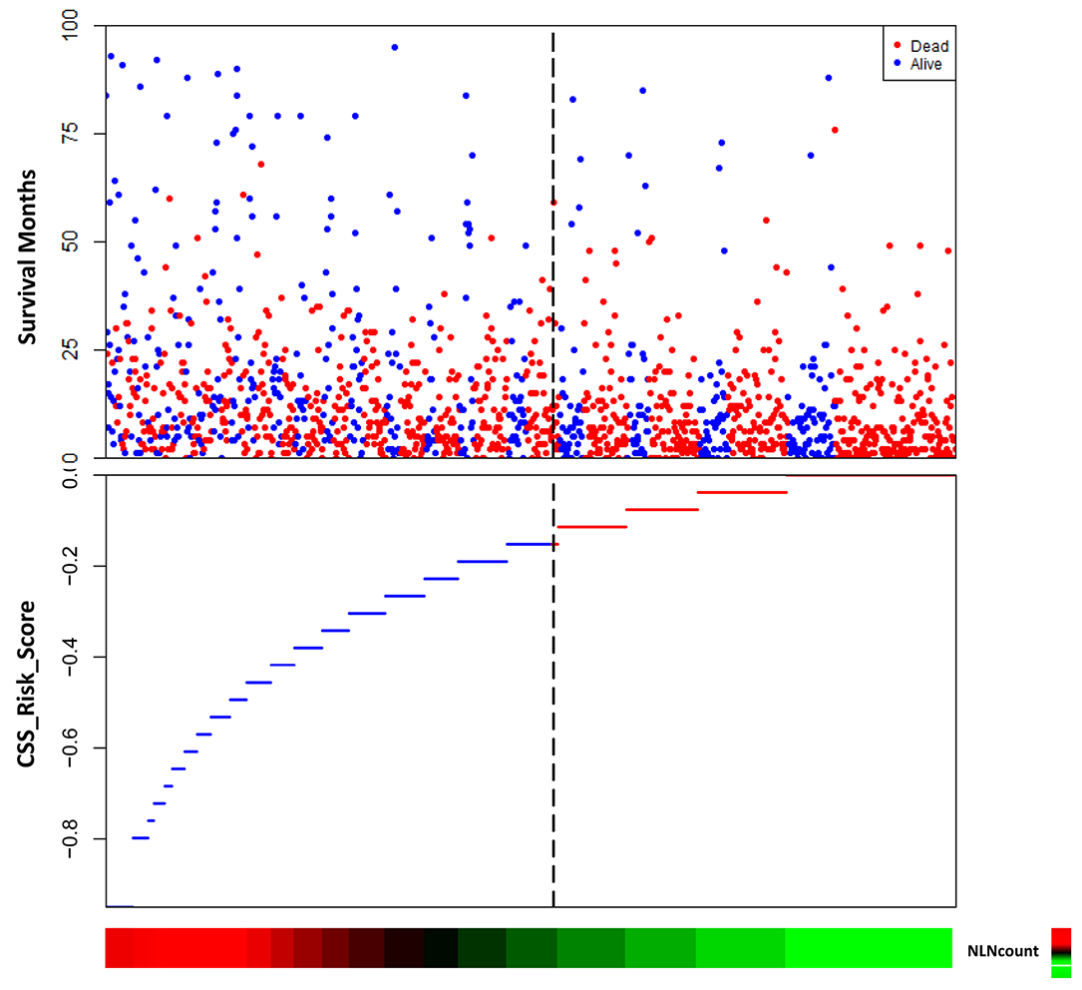
The risk score of NLN counts The risk score of negative lymph node (NLN) counts was built using the linear combination of NLN counts with the estimated regression coefficients. Distribution of death and survival status of the patients was shown. It showed that the plot of hazard ratios (HRs) for NLN counts increased sharply when the number of NLN counts decreased.

The risk score of NLN counts was also built using the linear combination of NLN counts with the estimated regression coefficients derived from abovementioned Cox regression analysis. It was studied as the weight to calculate the death risk score for each patient. Distribution of death and the survival status of the patients with stage IV gastric cancer was shown in Figure 3. It showed that the plot of HRs for NLN counts increased sharply when the number of NLN counts decreased.

### Further analysis of the prognostic value of NLN counts according to N stage

**Figure 4:**
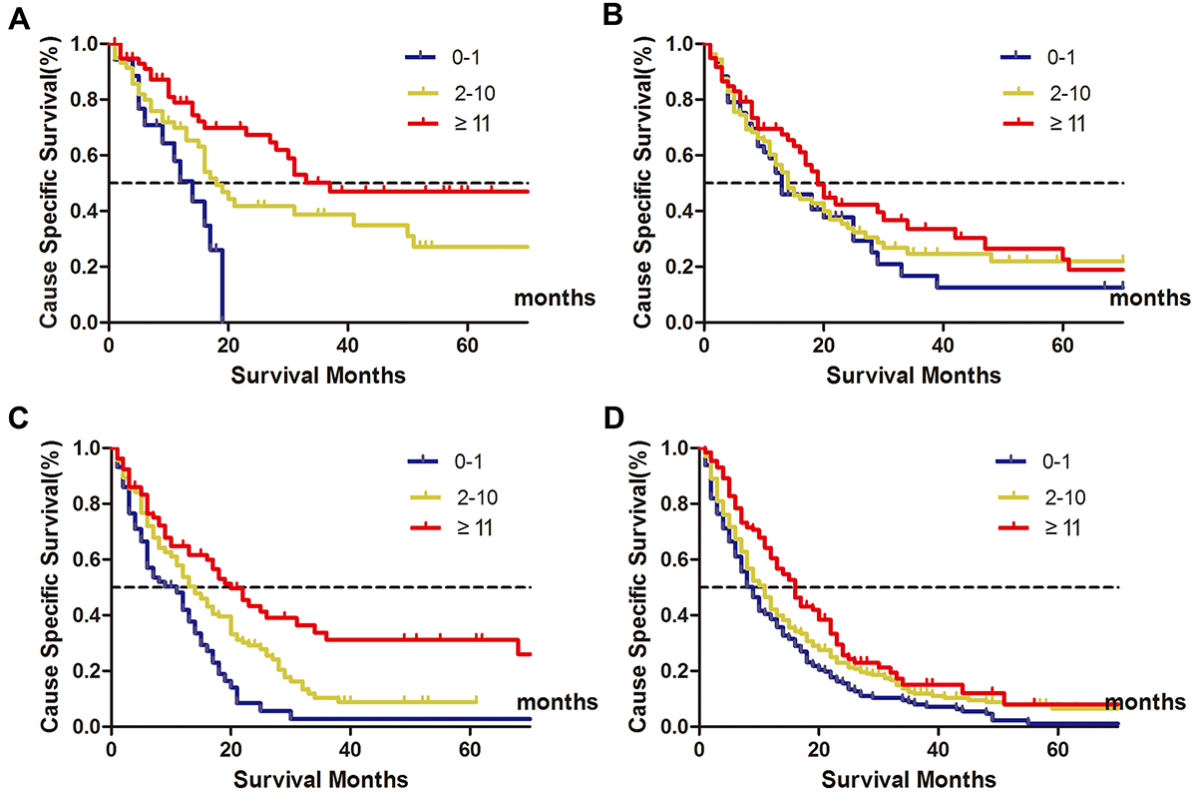
Clinical significance of NLN counts on patients’ survival stratified by N stage Subpopulation analysis of the prognostic value of negative lymph node (NLN) counts on patients’ survival according to N stage. For pN0 patients, *χ2*=10.615, *P*= 0.005 **(A)** For pN1 stage, *χ*2= 3.362, *P*= 0.186 **(B)** For pN2 stage, *χ*2= 26.503, P< 0.001 **(C)** For pN3 stage, *χ*2 = 22.509, *P* < 0.001 **(D)** It showed that the protective (more favorable) effect of higher NLN counts on patients’ 5-year cause-specific survival (CSS).

Since the N stage was one of the most powerful prognostic factors in stage I-III gastric cancer, we further made subgroup survival analysis to investigate the protective effect of higher NLN counts on patients’ 5-year CSS (Table 3). As it is shown, for pN0 patients, there was an absolute 46.3% increase in 5-year CSS when ≥11 NLNs were harvested compared to those who with 0-1 NLN (*P* < 0.030) (Figure 4A). For pN1 patients, although there also an increase in 5-year CSS when NLN counts increased, the difference was not significant (*P*= 0.186) (Figure 4B). For pN2 patients, the 5-year CSS for patients with 0-1, 2-10, ≥11 stage were 2.7%, 8.6% and 26.0%, respectively (*P*< 0.001), the 5-year CSS was nearly ten-folds in patients with ≥11 NLNs retrieved higher than that of 0-1 NLN (Figure 4C). For pN3 patients, the protective effect of NLN counts was also noticeable, the 5-year CSS for patients with 0-1, 2-10, ≥11 stage were 1.1%, 6.3% and 7.9% (Figure 4D).

**Table 3:** Univariate and multivariate analyses of NLN count on 5-year CSS based on different N stage

Subgroups			Univariate analysis		Multivariate analysis	
N stage	NLN counts	5-year CSS rate	*χ*^2^	*P*	HR (95% CI)	*P*
pN0			10.615	0.005		0.030
	0- 1	0			Reference	
	2- 10	25.9%			0.551 (0.277- 1.096)	0.089
	≥ 11	46.3%			0.371 (0.177- 0.775)	0.008
pN1			3.362	0.186		
	0- 1	12.0%				
	2- 10	20.1%				
	≥ 11	22.7%				
pN2			26.503	< 0.001		< 0.001
	0- 1	2.7%			Reference	
	2- 10	8.6%			0.652 (0.473- 0.898)	0.009
	≥ 11	26.0%			0.407 (0.269- 0.617)	< 0.001
pN3			22.509	< 0.001		< 0.001
	0- 1	1.1%			Reference	
	2- 10	6.3%			0.759 (0.630- 0.914)	0.004
	≥ 11	7.9%			0.566 (0.441- 0.727)	< 0.001

Abbreviation: N stage: lymph node status; NLN: negative lymph node; CSS: cause-specific survival; HR: hazard ratio; 95% CI: 95% confidence interval. Kaplan-Meier survival curves and Log-rank tests were used to identify the univariate risk factors. Cox regression model was used to perform multivariate analysis. Three subgroups were adjusted for age, race, and pathological grading. Histological type and T stage were treated as the covariates. *P*-value <0.05 was considered to be statistically significant.

## DISCUSSION

To the best of our knowledge, this is the first study to systematically evaluate the clinical significance of NLN counts in metastatic gastric cancer patients based on public available database. The results showed that increased NLNs count was an independently prognostic factor in stage IV gastric cancer after palliative resection. Also, the pathologic N stage is another predictor in stage IV gastric cancer. To be noted, the total LN number which was validated as a prognostic value in stage I-III gastric cancer patients lost its significance in this study.

The purpose of palliative gastrectomy is to relieve the symptoms associated with cancer, to prevent subsequent tumor complications, and to improve the quality of life of patients [13, 21, 22]. The palliative gastric resection is safe with low comorbidity and mortality. Results published after the mid-1990s revealed the mortality rates did not exceed 7% [11, 21]. In consideration of the surgical related complications, however, palliative resection was often done with limited LN dissection, such as D0 or D1 lymphadectomy. Up to one-third of patients with distant metastases undergo gastric resection without curative intent [23]. Considering the prognostic effect of N stage and NLN counts in surgical specimens revealed by the present study, accurate identification of positive lymph nodes and standard lymphadectomy should be considered essential for treatment.

In fact, the value of NLN counts was previously studied in patients with gastrointestinal carcinomas. But all patients in the published studies were received radical surgical resection [9, 17, 24]. Zhou *et al.* reported the clinical significance of primary tumor lymph node status in stage IV gastric cancer, they found lower positive lymph node ratio (LNR) was an independent predictor of longer survival in patients with gastric cancer after palliative resection [25], but they had not studied the value of NLN counts.

The protective effect of increased NLN retrieval in stage IV gastric cancer is very interesting. The tumor burden in distant metastases seems to be more serious than the tumor cells left in regional lymph node. However, it might be not the truth. The protective role of increased NLN count maybe related to cancer immunity. There were two kind of cancer immunity surrounding the tumor environment, namely antitumor immunity and immunological tolerance [26]. The latter one inclines to cause immune tolerance with the progress of the cancer development [27]. Resection of regional LNs might reset the immunological balance, resulting in an improvement of patients’ prognosis.

Although patients included in the present study under strictly selection, there were still some potential limitations. First, the SEER database lacks detailed therapy information. There has been great advance in palliative chemotherapy for gastric cancer nowadays. Patients received different and multi-sequential chemotherapy may cause the bias of this study. Second, the number of patients was not large enough. After stratification by N stage, there were limited numbers in each subgroups. This may weaken the statistical power. Third, since the absence of information of metastatic tumor burden and treatment on metastases, the exact survival benefit might be exaggerated due to possible simultaneous metastasectomy.

In conclusion, our present study revealed that lymph node status was still a strong prognostic predictor of survival for gastric cancer patients with palliative resection. NLN count was an independent risk factor in stage IV gastric cancer after palliative resection. Increased NLN retrieval can improve the patients’ 5-year CSS. Standard LN dissection, such as D2 lymphadectomy maybe still necessary during palliative resection for patients with metastatic gastric cancer.

## PATIENTS AND METHODS

### Patient characteristics

The clinical data of stage IV gastric cancer patients receiving palliative resection from January 2004 to December 2011 in SEER- registered database were retrieved. The SEER Program of the National Cancer Institute (NCI) is an authoritative source of information on cancer incidence and survival in the United States. SEER currently collects and publishes cancer incidence and survival data from population-based cancer registries covering 28 percent of the United Stated population [28, 29]. SEER*Stat Software Version 8.3.2 (NCI, USA) was used to download the database (https://seer.cancer.gov/seerstat/).

Only cases who fulfilled the following criteria were eligible for this study: (1) Years of diagnosis span from 2004 to 2011; (2) histological types were confined to adenocarcinoma, mucinous adenocarcinoma, and signet ring cell cancer; (3) gastric patients were confirmed with distant metastasis (M1); (4) patients received primary tumor resection; (5) with detailed information of tumor infiltration depth (T) stage, lymph node status (N) stage, and primary tumor size; (6) ages of patients studied were above 16; and (7) information on 5-year CSS and survival time was available. All patients were restaged according to UICC/AJCC TNM Stage system (7^th^ edition).

### Statistical analyses

The associations between N stage and clinicopathological features were analyzed using Chi-square test. Risk factors for survival outcomes were identified by Kaplan-Meier survival curves and Log-rank tests. Only variables that were significance in univariate analysis were included in the Cox multivariate regression model analysis. The X-tile program (http://www.tissuearray.org/rimmlab/) [30] which identified the cutoff with the minimum P values from log-rank *χ*2 statistics was used to divide patients into high, middle and low risk subsets, as previously described [31]. The primary endpoint of this study was 5-year CSS. Deaths attributed to gastric cancer were treated as events, while other reasons caused deaths or survivals were defined as censored events. All analyses were performed with survival package of R (Ver. 3.3.1) and SPSS (Ver. 22.0, IBM Corp., USA). Difference with *P*-value <0.05 was considered to be statistically significant.
